# Decoding CAPA: A Comparative Study of AspICU, ISHAM, and EORTC Criteria in Critical COVID-19 Patients Requiring Mechanical Ventilation

**DOI:** 10.3390/microorganisms14050978

**Published:** 2026-04-27

**Authors:** Chahnez Taleb, Christophe Lelubre, Patrick Biston, Michael Piagnerelli

**Affiliations:** 1 Intensive Care, Hopitaux Universitaires de Bruxelles, Université Libre de Bruxelles, 1070 Brussels, Belgium; 2 Internal Medicine, CHU (Centre Hospitalier Universitaire)-Charleroi Chimay, Université Libre de Bruxelles, 6042 Charleroi, Belgium; christophe.lelubre@humani.be; 3 Intensive Care, CHU (Centre Hospitalier Universitaire)-Charleroi Chimay, Université Libre de Bruxelles, 6042 Charleroi, Belgium; patrick.biston@humani.be (P.B.); michael.piagnerelli@humani.be (M.P.)

**Keywords:** COVID-19, pulmonary aspergillosis, CAPA, AspICU, ISHAM, EORTC, invasive fungal infections, critical care

## Abstract

COVID-19-associated pulmonary aspergillosis (CAPA) is a frequent and severe complication among critically ill patients with COVID-19. The absence of a clear diagnostic gold standard and the multiplicity of proposed definitions (EORTC/MSG, AspICU, and ISHAM) complicate its diagnosis. This study aimed to compare the performance of the EORTC/MSG, AspICU, and ISHAM classifications in diagnosing CAPA among mechanically ventilated COVID-19 patients and to assess their correlations with clinical outcomes. We conducted a retrospective, monocentric study including all adult COVID-19 patients requiring invasive mechanical ventilation admitted to the ICUs of CHU-Charleroi Chimay between March 2020 and December 2021. Patients were classified according to EORTC/MSG, AspICU, and ISHAM criteria. Demographics, comorbidities, management, and outcomes were compared across groups. In total, 405 patients were included during the four waves. The incidence of probable or possible CAPA varied widely: 6.1% with EORTC/MSG, 9.7% with AspICU, and 15.1% with ISHAM criteria. ICU mortality reached approximately 76% among patients with probable CAPA versus 43% in patients without aspergillosis. The frequency of CAPA diagnosis increased across COVID-19 waves, possibly correlating with changes in management, particularly corticosteroid use. The choice of CAPA diagnostic criteria has a major impact on incidence estimates and patient management. Prospective validation of CAPA definitions, integrating multiple mycological criteria, is urgently needed to guide clinical decision-making and antifungal therapy.

## 1. Introduction

The COVID-19 pandemic, caused by SARS-CoV-2, profoundly impacted the management of acute respiratory distress syndrome (ARDS) in the ICU [1]. Among its complications, COVID-19-associated pulmonary aspergillosis (CAPA) emerged as a significant threat, affecting up to 30% of ventilated patients [2]. Multiple risk factors predispose COVID-19 patients to CAPA, including direct viral damage to the respiratory epithelium, immune dysregulation, and prolonged mechanical ventilation [3]. Additionally, immunomodulatory treatments such as corticosteroids and IL-6 inhibitors further increase susceptibility [4,5,6].

Before COVID-19, the EORTC/MSG definitions were the reference for diagnosing invasive aspergillosis, primarily in immunocompromised patients [7]. However, these criteria are not well-suited for ICU patients, who often present atypical host factors and less specific radiological findings [2,8,9]. To address this gap, the AspICU algorithm was developed before the pandemic to facilitate the diagnosis of pulmonary aspergillosis in critically ill patients, allowing the definition of “putative” invasive disease based on positive respiratory cultures even in the absence of classical risk factors [10]. Following the pandemic, the ECMM/ISHAM consensus proposed adapted definitions for CAPA, with a stronger emphasis on mycological criteria [11].

Recent work, notably by Dellière et al. [12], provided additional insights into the diagnostic limitations of current definitions. In a retrospective cohort of 176 mechanically ventilated COVID-19 patients (with 350 respiratory samples analyzed), the authors systematically evaluated seven mycological criteria and highlighted that isolated positive results—such as a single BAL galactomannan or culture—were often non-reproducible and weakly predictive of outcomes. Conversely, the combination of multiple complementary tests, particularly those reflecting high fungal burden (direct microscopy, serum galactomannan, plasma Aspergillus qPCR), offered a more reliable approach for identifying clinically relevant infections. These findings underscore the current absence of a gold standard and the risk of misclassifying colonization as invasive disease.

Given this diagnostic uncertainty, it is crucial to evaluate the practical utility and prognostic relevance of existing CAPA definitions. Our objective was to assess and compare the EORTC/MSG, AspICU, and ISHAM classifications for CAPA in severe COVID-19 patients admitted to the ICU.

## 2. Methods

### 2.1. Study Design

We conducted a retrospective, observational, monocentric study including all COVID-19 patients admitted to the intensive care units (ICUs) of Centre Hospitalier Universitaire de Charleroi, Marie Curie and Vesale hospitals, Belgium, between March 2020 and December 2021. Approval was obtained from the local ethics committee (OM008), which waived the signed informed consent due to the retrospective design of the study.

### 2.2. Study Population

Inclusion criteria comprised an age of 18 years or older, a positive nasopharyngeal SARS-CoV-2 RT-PCR result, and the requirement for invasive mechanical ventilation. Patients were excluded if they were transferred from another hospital (due to incomplete data) or presented a positive PCR without clinical signs of lower respiratory tract infection (absence of fever, dyspnea, hypoxemia, and/or radiographic pulmonary infiltrates).

Collected data included demographic variables (age, sex, and body mass index), APACHE II and SOFA scores at ICU admission, comorbidities (hypertension, diabetes mellitus, chronic obstructive pulmonary disease/asthma, active cancer, chronic immunosuppression, and chronic kidney disease), as well as delays between symptom onset and ICU admission. For the purpose of this study, immunosuppression was defined as encompassing primary immunosuppressive conditions (e.g., haematological malignancy, solid organ transplantation) and chronic immunosuppressive therapies. Acute COVID-19-related immunomodulatory treatments—such as tocilizumab or short-term corticosteroids—were not classified as baseline immunosuppression and were analysed separately as management variables.

The majority of patients received dexamethasone at the standard COVID-19 protocol dosing (6 mg/day). Higher-dose corticosteroids (e.g., methylprednisolone) were administered in selected cases based on clinical severity and physician discretion; doses exceeding 2 mg/kg/day were not routinely prescribed in this cohort.

Management characteristics, including extracorporeal membrane oxygenation (ECMO) support, use of corticosteroids, and immunomodulators, including convalescent plasma from the CONFIDENT trial [9], were also recorded. Outcomes included length of ICU stay, ICU mortality, 28-day mortality and hospital mortality.

### 2.3. Diagnosis of CAPA

For microbiological investigations, screening for COVID-19-associated pulmonary aspergillosis (CAPA) included: serum and bronchoalveolar lavage (BAL) galactomannan detection, Aspergillus PCR, direct microscopic examination, and fungal culture from respiratory specimens. Investigations were prompted either by clinical/radiological deterioration or performed as part of routine screening protocols in ventilated patients. Sampling was performed at ICU admission or during clinical deterioration. Galactomannan thresholds followed institutional laboratory standards. Galactomannan positivity was interpreted according to local laboratory thresholds (optical density index ≥ 0.5 in serum and ≥1.0 in BAL), in line with commonly used clinical practice.

Patients were classified according to three diagnostic systems: the 2021 updated EORTC/MSG criteria [13], the AspICU algorithm [11], and the 2020 ECMM/ISHAM consensus definitions for CAPA [14]. To better compare the three diagnostic systems, we defined five internal clinical groups based on both microbiological findings and clinical context. The definition of each group is presented in Table 1.

### 2.4. Statistical Analysis: No Multivariate Analysis or Correction for Multiple Comparisons Was Performed, Given the Exploratory and Descriptive Nature of This Study

Descriptive statistics were computed. Quantitative variables are expressed as medians with interquartile ranges (IQR) or means ± standard deviation (SD), as appropriate. Qualitative variables are presented as absolute and relative frequencies. In comparative analyses on differences across groups and waves of COVID-19, categorical variables were compared using chi-squared tests or Fisher’s exact tests, and quantitative variables were compared using Mann–Whitney U tests or Kruskal–Wallis tests as appropriate. Modified scores were computed to facilitate two-by-two comparisons as explained before. The agreement between modified scores was displayed in bivariate scatterplots using disk areas proportional to the number of patients entering each combination. The degree of agreement between the modified ISHAM score and the modified EORTC score (whose response modalities were identical) was also computed using an unweighted kappa coefficient. Statistical analyses were performed on R version 4.4.2 (R Core Team (2024). _R: A Language and Environment for Statistical Computing. R Foundation for Statistical Computing, Vienna, Austria).

## 3. Results

### 3.1. Study Population

Among the 500 patients initially screened, 405 fulfilled the inclusion criteria. Demographic data are presented in Table 2. The median age was 63 years [IQR 54.8–71.0], with a male predominance (65.2%). The most prevalent comorbidities were hypertension (57.3%), diabetes mellitus (40.2%), chronic respiratory disease (20.7%), active malignancy (7.6%), and chronic immunosuppression (12.8%). The median body mass index was 29.1 [IQR 26.0–32.7] kg/m^2^. The median APACHE II score was 13 [IQR 10–17].

### 3.2. Respiratory Management and Outcomes

All 405 patients underwent invasive mechanical ventilation. Prone positioning was performed in 76% of patients, and ECMO support was used in 15.5% of cases (Table 3).

Overall ICU mortality was 47.2%, with the primary causes of death being refractory hypoxaemia and diffuse alveolar haemorrhage (Table 4).

### 3.3. CAPA Classification According to Diagnostic Criteria

According to EORTC/MSG criteria, probable CAPA was diagnosed in 4.9% of patients (n = 20), possible CAPA was diagnosed in 1.2% of patients (n = 5), and CAPA was ruled out in 93.8% of patients (n = 380).

According to the AspICU algorithm, probable/putative CAPA was identified in 8.5% (n = 34), colonization was identified in 2.1% (n = 9), and CAPA was ruled out in 89.3% of patients (n = 362).

According to the ECMM/ISHAM criteria, probable CAPA was found in 12.3% (n = 50), possible CAPA was found in 2.8% (n = 11), and CAPA was ruled out in 84.9% of patients (n = 344).

Detailed distribution of CAPA classification across COVID-19 waves is presented in Table 5 and Table 6.

Table 7 summarizes the use and results of mycological investigations across the entire cohort and according to the four COVID-19 waves. Overall, bronchoalveolar lavage (BAL) galactomannan (GM) testing was performed in 218 of 328 patients (66.5%), with an increasing testing rate over successive waves, reflecting evolving diagnostic practices. Among tested patients, serum GM positivity was observed at 14.7%, with a marked increase from the first wave (8.3%) to the fourth wave (21.7%). Median BAL GM index values also varied between waves, remaining generally low, with higher values observed during later waves.

Lower respiratory tract sampling was widely performed, with bronchoalveolar lavage (BAL) available in nearly all patients who underwent GM testing (99.0%). GM testing on sputum was rarely performed and showed very few positive results. Serum GM testing was less frequently performed, with a very low positivity rate overall (0.5%), reflecting its limited use and diagnostic yield in this population.

Aspergillus cultures from respiratory samples were positive in 9.8% of patients overall, with higher positivity rates observed during the third and fourth waves compared to earlier periods.

**Figure 1:**
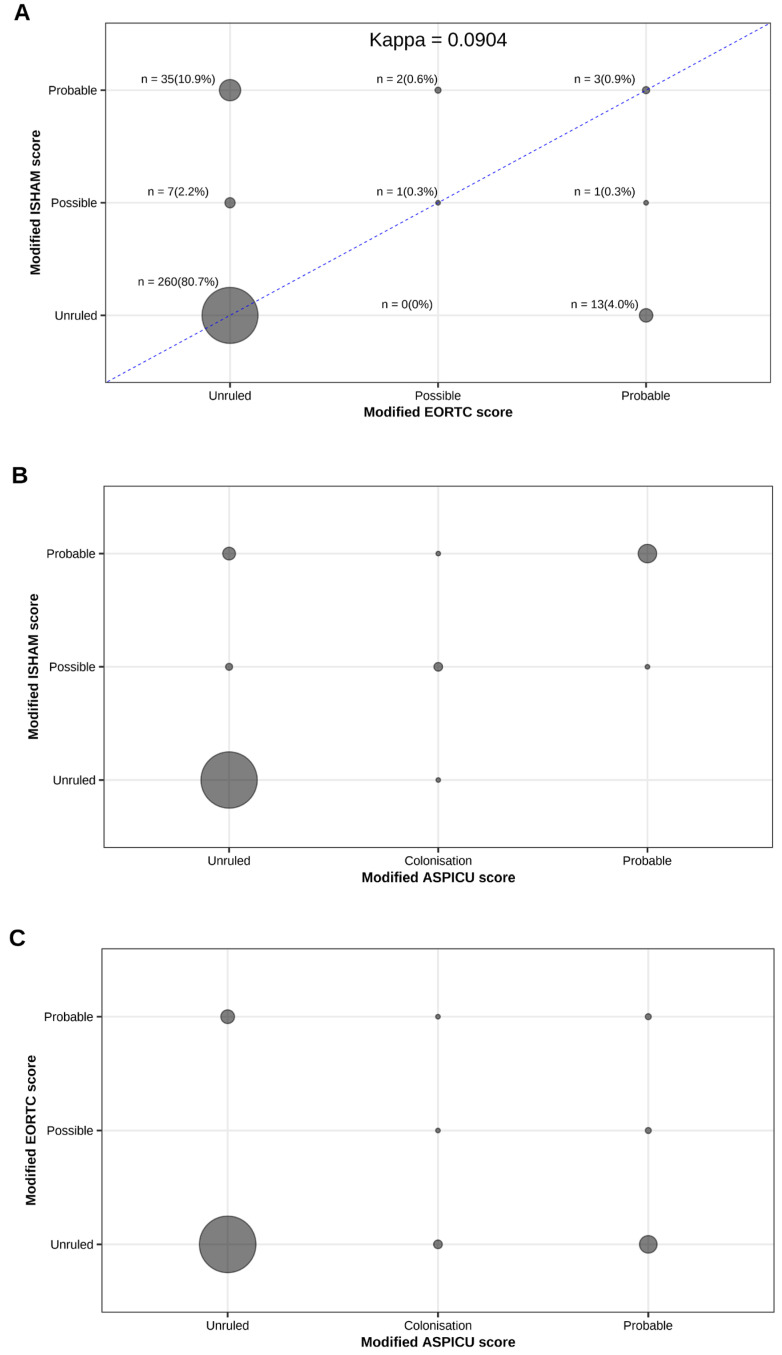
Concordance between the different classification systems. (**A**) Concordance between modified EORTC and modified ISHAM criteria. Circle size is proportional to the number of patients in each cell, with absolute numbers and percentages indicated. The diagonal line represents perfect agreement. The kappa coefficient is 0.09, indicating a low level of agreement between the two classifications. (**B**) Concordance between modified ISHAM and modified AspICU criteria. Circle size is proportional to the number of patients per cell. Most patients were classified as “unruled”, with some overlap observed in the “probable” and “possible” categories. (**C**) Concordance between modified EORTC and modified AspICU criteria. Circle size reflects the number of patients per cell. Most patients clustered in the “unruled” category, with some overlap in the “probable” and “possible” categories.

Figure 1 illustrates the concordance between the different classification systems used to diagnose CAPA, demonstrating a poor overall concordance between the three classification systems, particularly for intermediate and high-probability categories.

### 3.4. Mortality According to CAPA Classification

ICU mortality varied substantially according to the aspergillosis diagnosis group. Patients classified as probable CAPA (Group 3) showed a mortality of approximately 76%, compared with around 43% in patients without evidence of aspergillosis (Groups 0–1).

### 3.5. Evolution Across COVID-19 Waves

The incidence of CAPA increased progressively over successive waves, particularly when using the ECMM/ISHAM criteria. This trend correlated with changes in COVID-19 management practices, notably the introduction and generalization of corticosteroids following the RECOVERY trial results [15,16]. During the first wave, we observed a low incidence of CAPA, which coincided with the limited use of corticosteroids. The second wave was marked by a moderate increase in CAPA diagnoses, while the third and fourth waves showed a significant rise in probable CAPA cases, particularly according to the ECMM/ISHAM criteria.

## 4. Discussion

In this large cohort of critically ill COVID-19 patients requiring mechanical ventilation, we observed that the diagnosis of COVID-19-associated pulmonary aspergillosis (CAPA) remains highly dependent on the diagnostic criteria applied. The incidence of probable or possible CAPA ranged from 6.1% to 15.1%, depending on whether EORTC/MSG, AspICU, or ISHAM criteria were used.

The EORTC/MSG criteria [15], historically developed for classical immunocompromised patients (such as those with neutropenia or hematologic malignancies), appeared too restrictive for critically ill COVID-19 patients. Many cases of CAPA likely remained unidentified because these patients often lack the traditional host factors required for EORTC diagnosis. As a result, using EORTC criteria may lead to underdiagnosis of CAPA in ICU settings.

Conversely, the ISHAM consensus definitions [11] permitted a broader diagnosis by accepting isolated mycological criteria (such as a single positive BAL galactomannan or a positive culture) without mandatory host factors. While this approach increases sensitivity, it also risks overdiagnosing CAPA, including cases of simple Aspergillus colonization rather than true invasive disease. This highlights a major limitation of applying ISHAM definitions without integrating clinical context and additional microbiological corroboration.

The AspICU algorithm (11), although developed prior to the COVID-19 pandemic, may be more suitable for critically ill patients without classical immunosuppression. It aims to balance sensitivity and specificity by combining clinical deterioration with mycological and radiological findings (11). In our cohort, AspICU definitions tended to identify patients with a clinically meaningful infection burden, although some uncertainty remains regarding its ability to fully distinguish invasive disease from colonization in COVID-19 patients.

Some differences between classification systems come from how they define host risk factors. For example, immunosuppressed patients are considered to have probable aspergillosis under the EORTC/MSG criteria. However, when such patients lack positive mycological results, they do not meet the criteria for probable aspergillosis under either the AspICU or ISHAM frameworks. This discordance explains why an individual patient may be assigned to Group 0 under one system and Group 3 under another.

Galactomannan positivity was interpreted according to local laboratory thresholds (optical density index ≥ 0.5 in serum and ≥1.0 in BAL), in line with commonly used clinical practice. The increase in BAL galactomannan positivity during later waves may reflect both increased use of corticosteroids and immunomodulatory therapies, as well as more systematic screening strategies and improved clinical awareness of CAPA.

Empirical antifungal therapy was mainly initiated in patients with clinical deterioration and at least one positive mycological criterion (Group 3). Colonization cases were generally not treated unless clinical worsening occurred.

In our pragmatic classification, colonization (Group 1) refers to cases with a positive Aspergillus culture from the respiratory tract, but without any signs of respiratory worsening. Although this group included only a small number of patients, it nevertheless represents a clinically meaningful and pragmatically useful distinction.

Dellière et al. [12] conducted a one-year retrospective cohort study including 176 mechanically ventilated COVID-19 patients, with 350 respiratory samples analyzed to systematically assess seven mycological criteria for CAPA. They demonstrated that isolated positive findings—such as a single BAL galactomannan or a solitary positive culture—were often poorly reproducible and only weakly correlated with clinical outcomes, whereas combining several criteria, particularly those reflecting a high fungal burden (direct microscopy, serum galactomannan, or plasma Aspergillus qPCR), offered a more robust diagnostic approach and clearer prognostic value. This work underlines the importance of integrating multiple complementary tests rather than relying on a single positive result to enhance diagnostic precision and minimize the risk of misclassifying colonization as invasive disease.

Importantly, patients classified as probable CAPA—regardless of the definition used—had significantly higher ICU mortality. This observation reinforces the clinical relevance of CAPA and suggests that at least a proportion of diagnosed cases reflect true invasive infection rather than colonization. However, these findings do not necessarily imply a causal relationship between excess mortality and CAPA diagnosis, given the well-established multifactorial nature of mortality in severe COVID-19. CAPA could increase the risk of poor outcomes or represent a marker of disease severity, rather than being the direct cause of death. Mortality comparisons presented are unadjusted and should be interpreted cautiously.

Our study has several strengths, including a large cohort size, systematic application of three major diagnostic definitions, and analysis across successive COVID-19 waves.

However, some limitations must be acknowledged. The retrospective, monocentric design may limit external generalizability. All patients were consecutively included, and Aspergillus investigations were performed either systematically during later waves or when clinically suspected, which may have introduced selection bias. Serum galactomannan testing was not routinely performed outside immunocompromised patients, which might have underestimated CAPA diagnoses by ISHAM criteria. Radiological assessment was not standardized due to logistical challenges during the pandemic. Finally, our adaptation of CAPA classifications into a five-group system (from 0 to 4) was an arbitrary simplification intended to facilitate comparison across criteria. Although useful for standardization, this approach may not perfectly reflect the nuances of each original definition.

Overall, our findings highlight the urgent need for a pragmatic, harmonized approach to CAPA diagnosis, tailored to the ICU setting. Prospective validation of combined diagnostic criteria could help optimize antifungal therapy decisions and improve patient outcomes.

## 5. Conclusions

Diagnosing COVID-19-associated pulmonary aspergillosis (CAPA) in critically ill patients requiring mechanical ventilation remains a major challenge, particularly in the absence of a clear diagnostic gold standard. Our study highlights the significant variability in CAPA incidence depending on the diagnostic criteria applied. While EORTC/MSG criteria appear too restrictive for ICU patients, ISHAM definitions may lead to overdiagnosis. The AspICU algorithm offers a pragmatic alternative better suited to critically ill, non-classically immunocompromised patients. Given the high mortality associated with probable CAPA cases, future prospective studies should aim to refine CAPA definitions and integrate multiple mycological criteria to improve diagnostic accuracy in the ICU setting.

## Figures and Tables

**Table 1:** Internal Clinical Classification of CAPA Suspicion and Diagnosis in ICU Patients.

Group 0—No suspected aspergillosis: No mycological analysis was performed during ICU stay, as patients remained stable or their respiratory condition improved throughout their course. Group 1—Aspergillus colonization: Defined by a positive respiratory Aspergillus culture in a patient with no signs of respiratory, hemodynamic deterioration, or persisting fever. Group 2—Possible aspergillosis: Defined by the onset of respiratory or hemodynamic worsening and/or persistent fever prompting a mycological investigation (BAL, tracheal aspirate, etc.), regardless of whether the result was positive or negative. Group 3—Probable aspergillosis: Patients presenting with clinical deterioration and at least one positive mycological test (culture or galactomannan) consistent with invasive fungal disease. Group 4—Proven aspergillosis: Confirmed by anatomopathological evidence of tissue invasion by Aspergillus species

**Table 2:** Characteristics of ICU Patients by COVID-19 Wave.

Characteristic	Overall N = 328	1, N = 25	2, N = 105	3, N = 135	4, N = 63
Age	63.0 (54.8, 71.0)	60.0 (56.0, 66.0)	66.0 (59.0, 73.0)	64.0 (57.0, 70.0)	58.0 (50.0, 66.5)
Females	114/328 (34.8%)	10/25 (40.0%)	36/105 (34.3%)	43/135 (31.9%)	25/63 (39.7%)
Males	214/328 (65.2%)	15/25 (60.0%)	69/105 (65.7%)	92/135 (68.1%)	38/63 (60.3%)
BMI	30.1 (26.8, 34.5)	30.4 (28.7, 33.3)	29.8 (26.7, 34.3)	30.3 (26.5, 33.5)	31.0 (27.0, 37.0)
Comorbidities					
Hypertension	188/328 (57.3%)	19/25 (76.0%)	66/105 (62.9%)	70/135 (51.9%)	33/63 (52.4%)
Diabetes	132/328 (40.2%)	10/25 (40.0%)	46/105 (43.8%)	47/135 (34.8%)	29/63 (46.0%)
Ischemic heart disease	41/328 (12.5%)	2/25 (8.0%)	10/105 (9.5%)	22/135 (16.3%)	7/63 (11.1%)
Immunosuppression	42/328 (12.8%)	2/25 (8.0%)	11/105 (10.5%)	13/135 (9.6%)	16/63 (25.4%)
Chronic kidney failure	42/328 (12.8%)	2/25 (8.0%)	12/105 (11.4%)	20/135 (14.8%)	8/63 (12.7%)
COPD	34/328 (10.4%)	2/25 (8.0%)	17/105 (16.2%)	13/135 (9.6%)	2/63 (3.2%)
Active cancer (<2 years)	22/328 (6.7%)	0/25 (0.0%)	11/105 (10.5%)	9/135 (6.7%)	2/63 (3.2%)
SOFA score at ICU admission	5.0 (3.0, 7.0)	6.0 (4.0, 7.0)	5.0 (4.0, 8.0)	4.0 (3.0, 7.0)	5.0 (4.0, 7.0)
APACHE score	13.0 (10.0, 17.0)	13.0 (10.0, 19.0)	15.0 (11.0, 18.0)	12.0 (9.0, 16.0)	10.0 (9.0, 14.0)

**Table 3:** Respiratory support and medications during ICU stay.

Characteristic	Overall N = 328	1, N = 25	2, N = 105	3, N = 135	4, N = 63
Mechanical ventilation duration (days)	12.0 (6.0, 20.0)	10.0 (6.0, 14.0)	10.0 (6.0, 16.0)	15.0 (8.0, 24.5)	13.0 (6.0, 21.0)
Sedation duration (days)	11.0 (5.0, 16.0)	7.0 (4.0, 12.0)	8.5 (5.0, 13.0)	12.0 (7.0, 17.8)	11.5 (6.0, 19.3)
Prone position (session)	2.0 (1.0, 3.0)	2.0 (1.0, 2.0)	1.0 (1.0, 3.0)	2.0 (1.0, 3.0)	2.5 (1.0, 6.0)
Days under curare use	8.0 (4.0, 14.0)	5.0 (3.0, 10.0)	6.0 (2.0, 10.0)	10.0 (5.0, 15.0)	11.0 (5.8, 18.0)
ECMO support	51/328 (15.5%)	6/25 (24.0%)	14/105 (13.3%)	24/135 (17.8%)	7/63 (11.1%)
ECMO days to weaning (days)	13.0 (8.0, 20.5)	10.5 (7.3, 18.3)	9.5 (8.0, 12.8)	19.0 (12.0, 28.0)	12.0 (6.0, 25.0)
Use of dexamethasone	290/327 (88.7%)	0/25 (0.0%)	99/105 (94.3%)	131/134 (97.8%)	60/63 (95.2%)
Methylprednisolone	36/326 (11.0%)	0/25 (0.0%)	1/105 (1.0%)	21/133 (15.8%)	14/63 (22.2%)
Hydrocortisone	22/327 (6.7%)	21/25 (84.0%)	1/105 (1.0%)	0/135 (0.0%)	0/62 (0.0%)
Azithromycin	7/327 (2.1%)	2/25 (8.0%)	2/105 (1.9%)	2/135 (1.5%)	1/62 (1.6%)
Remdesivir use	6/326 (1.8%)	0/25 (0.0%)	6/105 (5.7%)	0/134 (0.0%)	0/62 (0.0%)
Convalescent plasma	35/326 (10.7%)	0/25 (0.0%)	22/105 (21.0%)	13/135 (9.6%)	0/61 (0.0%)

**Table 4:** COVID symptoms and survival.

Characteristic	Overall N = 328	1, N = 25	2, N = 105	3, N = 135	4, N = 63
Previous COVID symptoms (days)	8.0 (6.0, 10.0)	9.0 (5.0, 12.0)	8.0 (6.3, 10.0)	8.0 (7.0, 10.0)	8.0 (6.0, 10.0)
ICU ospitalization from symptoms (days)	1.0 (0.0, 3.0)	0.0 (0.0, 2.0)	1.0 (0.0, 2.0)	1.0 (0.0, 3.0)	1.0 (0.0, 4.0)
ICU LOS (days)	14.0 (8.0, 23.0)	14.0 (10.0, 20.0)	12.0 (7.0, 19.0)	16.0 (10.0, 26.8)	13.0 (7.5, 23.5)
Hospital LOS (days)	21.0 (13.5, 33.5)	21.0 (14.0, 29.0)	19.0 (11.0, 27.0)	23.0 (15.0, 36.8)	23.5 (13.8, 40.3)
ICU survival	192/328 (58.5%)	17/25 (68.0%)	56/105 (53.3%)	88/135 (65.2%)	31/63 (49.2%)
Survival at day 28	198/327 (60.6%)	17/25 (68.0%)	57/105 (54.3%)	90/134 (67.2%)	34/63 (54.0%)
Hospital survival	183/328 (55.8%)	17/25 (68.0%)	55/105 (52.4%)	81/135 (60.0%)	30/63 (47.6%)

**Table 5:** Microbiologic testing of all patients according to each wave.

Characteristic	Overall N = 218	1, N = 25	2, N = 105	3, N = 135	4, N = 63
Positive galactomannan	32/218 (14.7%)	1/12 (8.3%)	6/75 (8.0%)	15/85 (17.6%)	10/46 (21.7%)
Galactomannan result	0.2 (0.1, 1.5)	6.5 (6.5, 6.5)	2.0 (1.3, 3.1)	0.1 (0.1, 0.4)	2.4 (0.5, 2.6)
Galactomanan dosing—sputum	1/206 (0.5%)	0/12 (0.0%)	0/73 (0.0%)	1/84 (1.2%)	0/37 (0.0%)
LBA galactomannan dosing	204/206 (99.0%)	12/12 (100.0%)	73/73 (100.0%)	83/84 (98.8%)	36/37 (97.3%)
Serum galactomannan dosing	1/206 (0.5%)	0/12 (0.0%)	0/73 (0.0%)	0/84 (0.0%)	1/37 (2.7%)
Positive aspergillus culture in sputum	32/328 (9.8%)	1/25 (4.0%)	3/105 (2.9%)	18/135 (13.3%)	10/63 (15.9%)

**Table 6:** Diagnosis of CAPA according to EORTC, ISHAM, and AspICU criteria through waves and overall data.

EORTC	Overall	Wave 1	Wave 2	Wave 3	Wave 4
Ruled out	304/324 (93.8%)	23/25 (92.0%)	98/104 (94.2%)	132/135 (97.8%)	51/60 (85.0%)
Possible aspergillosis	16/324 (4.9%)	2/25 (8.0%)	6/104 (5.8%)	2/135 (1.5%)	6/60 (10.0%)
Probable aspergillosis	4/324 (1.2%)	0/25 (0.0%)	0/104 (0.0%)	1/135 (0.7%)	3/60 (5.0%)
ASPICU					
Ruled out	293/328 (89.3%)	24/25 (96.0%)	103/105 (98.1%)	114/135 (84.4%)	52/63 (82.5%)
Colonization	7/328 (2.1%)	0/25 (0.0%)	1/105 (1.0%)	5/135 (3.7%)	1/63 (1.6%)
Putative	25/328 (7.6%)	1/25 (4.0%)	1/105 (1.0%)	16/135 (11.9%)	7/63 (11.1%)
Probable	3/328 (0.9%)	0/25 (0.0%)	0/105 (0.0%)	0/135 (0.0%)	3/63 (4.8%)
ISHAM					
Ruled out	275/324 (84.9%)	24/25 (96.0%)	96/105 (91.4%)	110/135 (81.5%)	45/59 (76.3%)
Possible	9/324 (2.8%)	0/25 (0.0%)	2/105 (1.9%)	4/135 (3.0%)	3/59 (5.1%)
Probable	40/324 (12.3%)	1/25 (4.0%)	7/105 (6.7%)	21/135 (15.6%)	11/59 (18.6%)

**Table 7:** Diagnosis of CAPA according to our recoding of EORTC, ISHAM, and AspICU criteria through waves and overall data.

Recoded EORTC	Overall	Wave 1	Wave 2	Wave 3	Wave 4
0	305/325 (93.8%)	23/25 (92.0%)	99/105 (94.3%)	132/135 (97.8%)	51/60 (85.0%)
2	3/325 (0.9%)	0/25 (0.0%)	0/105 (0.0%)	0/135 (0.0%)	3/60 (5.0%)
3	17/325 (5.2%)	2/25 (8.0%)	6/105 (5.7%)	3/135 (2.2%)	6/60 (10.0%)
Recoded AspICU criteria					
0	293/328 (89.3%)	24/25 (96.0%)	103/105 (98.1%)	114/135 (84.4%)	52/63 (82.5%)
1	7/328 (2.1%)	0/25 (0.0%)	1/105 (1.0%)	5/135 (3.7%)	1/63 (1.6%)
3	28/328 (8.5%)	1/25 (4.0%)	1/105 (1.0%)	16/135 (11.9%)	10/63 (15.9%)
Recoded ISHAM criteria					
0	275/324 (84.9%)	24/25 (96.0%)	96/105 (91.4%)	110/135 (81.5%)	45/59 (76.3%)
2	9/324 (2.8%)	0/25 (0.0%)	2/105 (1.9%)	4/135 (3.0%)	3/59 (5.1%)
3	40/324 (12.3%)	1/25 (4.0%)	7/105 (6.7%)	21/135 (15.6%)	11/59 (18.6%)

## Data Availability

The data presented in this study are available on request from the corresponding author. The data are not publicly available due to privacy.
